# Bibliometric Analysis of the Mental Health of International Migrants

**DOI:** 10.3390/ijerph22081187

**Published:** 2025-07-29

**Authors:** Lei Han, Seunghui Jeong, Seongwon Kim, Yunjeong Eom, Minye Jung

**Affiliations:** 1 Department of Occupational Therapy, Graduate School, Yonsei University, Wonju 26493, Republic of Korea; lhan@yonsei.ac.kr (L.H.); wjdtmdgml98@yonsei.ac.kr (S.J.); kswon94@yonsei.ac.kr (S.K.); uhmazing@yonsei.ac.kr (Y.E.); 2 Department of Occupational Therapy, College of Software and Digital Healthcare Convergence, Yonsei University, Wonju 26493, Republic of Korea

**Keywords:** mental health, international migrants, migrant workers, international students, refugees, asylum seekers, smuggled migrants, bibliometric analysis

## Abstract

Background: International migration is a growing global phenomenon involving diverse groups, such as labor migrants, international marriage migrants, refugees, and international students. International migrants face unique mental health challenges influenced by adversities such as social isolation and limited access to mental health services. This study employs bibliometric methods to systematically analyze the global body of literature on international migrants’ mental health. Methods: The literature on the mental health of international migrants published until October 2024 was searched using the Web of Science database. The search terms included (‘International migrants’ OR ‘migrant workers’ OR ‘international students’ OR ‘refugees’ OR ‘asylum seekers’ OR ‘smuggled migrants’) AND ‘mental health’. VOSviewer was used to conduct bibliometric analysis, focusing on co-authorship patterns, keyword co-occurrence, and citation networks. Results: Over the past four decades, research on the mental health of international migrants has grown substantially, with major migration destinations such as the United States, Europe, and Australia playing prominent roles in this field. ‘Post-traumatic stress disorder (PTSD)’ was the most frequent keyword in publications, with strong links to ‘trauma’ and ‘depression’. In recent years, with the impact of global socioenvironmental changes and emergencies such as the COVID-19 pandemic, the research focus has gradually shifted towards social support, service accessibility, and cultural adaptation. Conclusions: International migration is a far-reaching global phenomenon, and addressing the mental health of migrant populations is essential for advancing public health, social cohesion, and sustainable development. This study provides the first bibliometric overview of research in this domain, mapping its thematic evolution and collaborative structure. The findings offer valuable insights into the field’s development and may support future interdisciplinary collaboration and the formulation of culturally informed, evidence-based approaches in migrant mental health.

## 1. Introduction

According to the United Nation’s International Organization for Migration (IOM), an international migrant is any individual who is moving or has moved away from their habitual place of residence across borders, regardless of their legal status, motives for migration, voluntariness, or duration of stay. Consequently, the term ‘international migrant’ is extensive in scope, encompassing labor migrants, family reunification migrants, refugees, asylum seekers, individuals migrating due to environmental or climate-related factors, and international students, among other diverse groups [1]. According to the World Migration Report 2024, as of 2022, there were approximately 281 million international migrants globally, constituting 3.6% of the world’s total population, and this trend continues to rise [2].

Alongside the increase in global migration, mental health has become a critical concern for international migrants. The 2022 World Report on the Health of Refugees and Migrants by the World Health Organization (WHO) highlighted that international migrants face a range of mental health challenges, including anxiety, depression, and post-traumatic stress disorder (PTSD), which often occur at higher rates than those among the local populations of host countries [3]. The report indicated that international migrants’ mental health is influenced by various factors such as adversities encountered during migration, social isolation, and instability of legal status, all of which can contribute to the development of mental health disorders [4]. Additionally, international migrants often encounter barriers when seeking mental health services, such as limited awareness of available services, language difficulties, and concerns about social exclusion, which further exacerbate their mental health risks [5]. Mental health disorders have detrimental effects on daily life, social participation, and career development [6,7].

The 2030 Agenda for Sustainable Development, adopted by the United Nations General Assembly in 2015, explicitly integrated migration into core development considerations, marking the first time that migration was formally included in a global development agenda [8]. At the high-level meeting of the Third Global Consultation on the Health of Refugees and Migrants, the Rabat Declaration aimed at improving the health of refugees and migrants reaffirmed that everyone, including refugees and migrants, has the right to the highest attainable standard of physical and mental health [9]. In this context, the 2022 World Report by the World Health Organization (WHO) reiterated the necessity of multidisciplinary interventions to address the mental health needs of migrant populations [10,11], which align directly with the United Nations Sustainable Development Goals (SDGs), particularly Goal 3 (“Good Health and Well-being”) and Goal 10 (“Reduced Inequalities”) [8].

Although growing attention is being paid to the mental health issues of migrants, including mental health assessments, intervention strategies, and policy impacts, most studies have primarily focused on identifying psychological disorders in migrants, with less emphasis on exploring effective intervention measures and treatment options [4,12]. Moreover, although there are some quantitative analyses of the literature on migrant health, the comprehensive and systematic review of the research development in the field of migrant mental health is still limited. To better understand the current state of research, map research trends and directions, and provide theoretical guidance for future research and clinical practice in related disciplines, it is necessary to conduct a comprehensive review and synthesis of the existing literature.

Bibliometrics is a discipline that studies the quantity and characteristics of the literature. It evaluates the output and dynamics of academic research through a quantitative analysis of the production, citation, and influence of the literature [13]. It is generally used to identify research hotspots in disciplines, analyze research collaboration networks, and track the evolution of research trends [14]. The unique strength of bibliometrics is its ability to provide data support and reveal key factors that influence the development of research topics. By analyzing the frequently cited literature and author networks, bibliometrics provides researchers and policymakers with insights to better understand and plan future research directions and facilitates interdisciplinary collaboration [15,16].

This study employs bibliometric methods to systematically analyze the global body of literature on international migrants’ mental health. Its purpose is to clarify key research domains, thematic trends, and patterns of scientific collaboration over the past four decades. The analysis aims to provide an evidence-based overview to support future interdisciplinary research, promote mental health equity, and inform policy and practice. Specifically, the study addresses the following questions: What have been the primary areas of focus in migrant mental health research? What trends and hotspots have emerged? Which international collaborations and knowledge networks can be identified? And what research gaps or challenges require further exploration?

## 2. Materials and Methods

### 2.1. Data Collection

The Web of Science (WoS) was used as the data source to retrieve relevant articles. The WoS is a widely used and authoritative international academic database, known for its high-quality bibliometric data, lower rate of duplicate records, and extensive coverage of high-impact journals [17,18]. It provides comprehensive and structured metadata, including article titles, authors, affiliations, countries or regions, publication years, and keywords, which are essential for bibliometric research [19]. In addition, the WoS is fully compatible with commonly used bibliometric tools such as VOSviewer, making it particularly suitable for bibliometric analyses [20].

The literature search was conducted for articles published up to October 2024. To search for publications that were closest matches, a search strategy was established based on each component of the definition of international migrants and with reference to published review literature [21,22,23,24,25]. The following search string was used: (‘international migrants’ OR ‘migrant workers’ OR ‘international students’ OR ‘refugees’ OR ‘asylum seekers’ OR ‘smuggled migrants’) AND ‘mental health’. Papers were selected according to the inclusion criteria, and relevant information was extracted. This study included all articles with abstracts and excluded news, congresses, and letters to the editor. The data was exported in the CSV format for further analysis.

### 2.2. Data Analysis and Visualization Maps

This study utilized a bibliometric analysis to systematically evaluate global scholarly output on the mental health of international migrants. Bibliometric analysis is a set of methods employed to quantitatively analyze the academic literature by applying mathematical and statistical tools [26]. This method provides a comprehensive understanding of the research trends, networks, and major thematic areas related to the mental health of various migrant groups.

To assess publication trends over time, annual publication counts were extracted from the publication year metadata provided in the WoS. Research fields were defined based on the “Web of Science Categories” assigned to each publication in the WoS database. The top ten categories were identified based on frequency to provide insight into the dominant disciplinary domains within the field.

The VOSviewer package (version 1.6.20.0) was used for comprehensive bibliometric analysis, including country, institution, author, keyword, and citation analyses. The analysis of countries, regions, and research institutions elucidated the global networks of research collaboration as well as their impact and contributions, while co-author analysis mapped the network of collaboration between authors. Keyword co-occurrence analysis examined the frequency and conceptual relationships among terms appearing in selected articles. A minimum threshold of 10 keyword occurrences was applied to enhance thematic relevance. Clusters were identified using VOSviewer’s default modularity-based clustering algorithm, with each cluster visualized in a distinct color representing major thematic domains. We investigated citation relationships through citation analysis to determine the impact of articles. Additionally, the application of two standard weight attributes, ‘link attribute’ and ‘total link strength attribute’ [27], provided nuanced insights into the relationships and salience of individual nodes in a network.

## 3. Results

### 3.1. Publication Output

**Figure 1:**
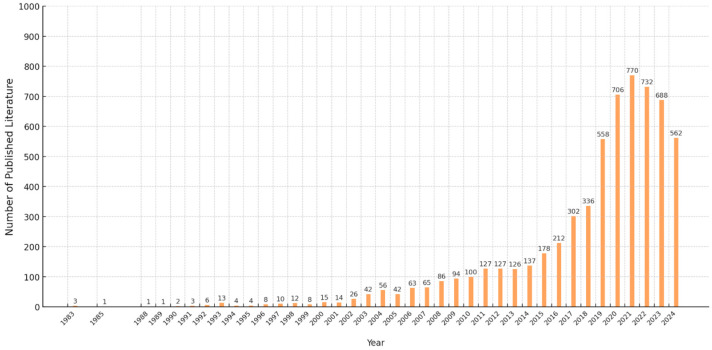
Number of published articles on mental health of international migrants by year.

**Figure 2:**
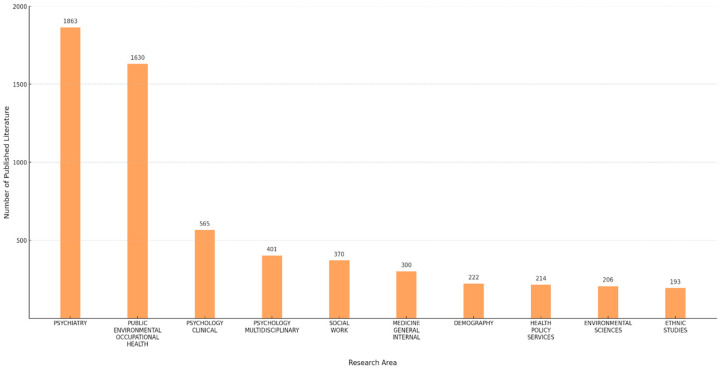
Top 10 research areas on the mental health of international migrants.

A total of 6240 publications were analyzed in this study. The research output began with a single paper in 1983 and escalated to 100 by 2010, marking the first instance of more than 100 publications in a single year. Since then, there has been a gradual increase in the number of publications each year, particularly in the last decade, with a steady growth trend in the number of annual publications on the subject (Figure 1). The highest number of publications was recorded in 2021, with 770. Among the 6240 publications on the mental health of international migrants, the top ten research fields are shown in Figure 2. Psychiatry ranked first with 1863 publications, followed by 1630 publications in public environmental occupations, 565 in clinical psychology, 401 in multidisciplinary psychology, and 370 in social work. The 5th through 10th were general internal medicine, demographics, health policy services, environmental sciences, and ethnic studies.

### 3.2. Distribution and Co-Authorship of Countries and Regions

**Figure 3:**
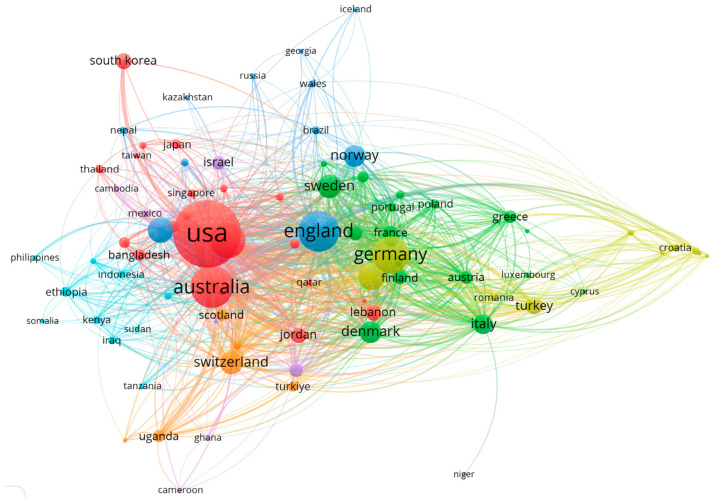
Distribution and Co-Authorship of Countries and Regions.

Based on the search results, 6240 publications were obtained from 142 countries and regions. As shown in Table 1, the United States had the largest number of publications (2096/6240), followed by Australia (851/6240) and the United Kingdom (778/6240). Figure 3 shows the locations of the 142 countries and regions that published research on the mental health of international migrants. A co-authorship analysis of countries and regions reflected their relationship with the degree of collaboration in the field. Larger nodes represent more productive countries and regions. The thickness and length of the links between nodes represent cooperative relationships between countries and regions. The 142 countries and regions from the seven collaboration clusters are distinguished by different colors.

### 3.3. Distribution and Co-Authorship of Organizations

**Figure 4:**
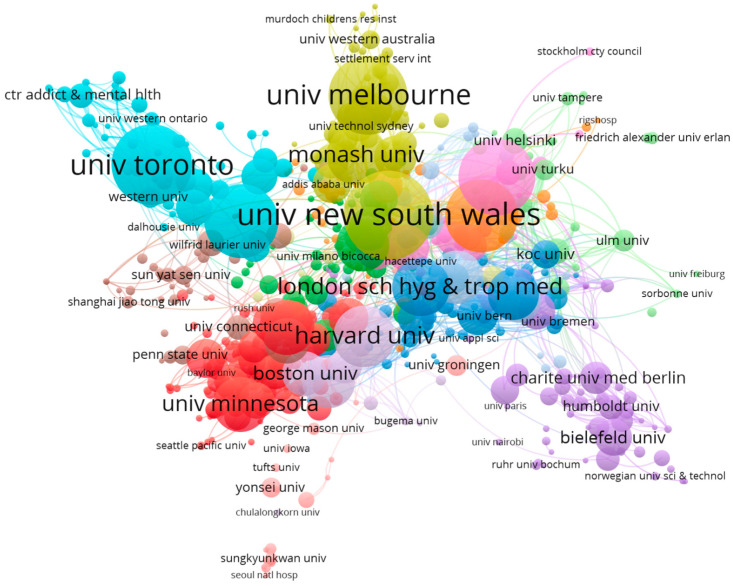
Distribution and co-authorship of organizations.

According to the search results, 5581 research organizations have contributed to research on the mental health of international migrants. Table 2 presents the 5 most productive organizations in related research. The University of New South Wales (161 publications) ranked first among all the identified organizations, followed by Toronto University (142 publications), Melbourne University (137 publications), Karolinska Institutet (133 publications), and McGill University (127 publications). Co-authorship analysis was performed using VOSviewer to display a visualization network map of organizations in international migrants’ mental health research. The links between institutions were determined by the number of co-authored publications, each of which published at least five papers and formed 14 clusters. These clusters are shown in Figure 4.

### 3.4. Distribution of Source Journals

Table 3A lists the top 10 journals that published research on this topic. The International Journal of Environmental Research and Public Health published the most articles (189/6240), followed by the Journal of Immigrant and Minority Health (172/6240) and BMC Public Health (131/6240). The top 10 journals published 1125 publications, accounting for 18.02% of all publications in this study.

We also performed citation analysis of the journals; the highest number of citations was found in JAMA—Journal of the American Medical Association (6487 citations), followed by The Journal of Nervous and Mental Disease (5271 citations), and Social Science & Medicine (4742 citations). Table 3B lists the top 10 journals with most citations on the subject.

### 3.5. Distribution and Co-Authorship of Authors

**Figure 5:**
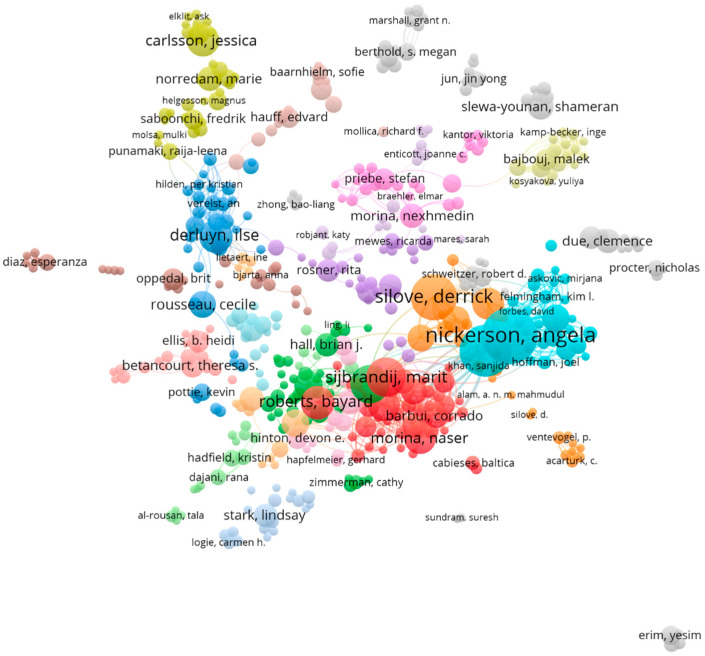
Distribution and co-authorship of authors.

Table 4 presents the top 10 most productive authors in research on the mental health of international migrants. Angela Nickerson (73 publications) from the School of Psychology at the University of New South Wales, Australia, has published the most articles in this field, ranking first, followed by Richard A. Bryant (66 publications) and Derrick Silove (56 publications). Based on the analysis of citations, Derrick Silove (3762) ranked first, while Richard A. Bryant (3618) and Zachary Steel (3048) secured the second and third positions, respectively. These clusters are shown in Figure 5.

### 3.6. Co-Occurrence Analysis of Top Keywords and Themes

VOSviewer was employed to extract and cluster the top 200 keywords, excluding the search terms such as ‘mental health’, ‘international migrants’ OR ‘migrant workers’ OR ‘international students’ OR ‘refugees’ OR ‘asylum seekers’ OR ‘smuggled migrants’. Table 5 displays the frequencies and link strengths of the top 10 keywords, with ‘PTSD (Post-Traumatic Stress Disorder)’, ‘trauma’, and ‘depression’ emerging as the most frequently occurring terms.

**Figure 6:**
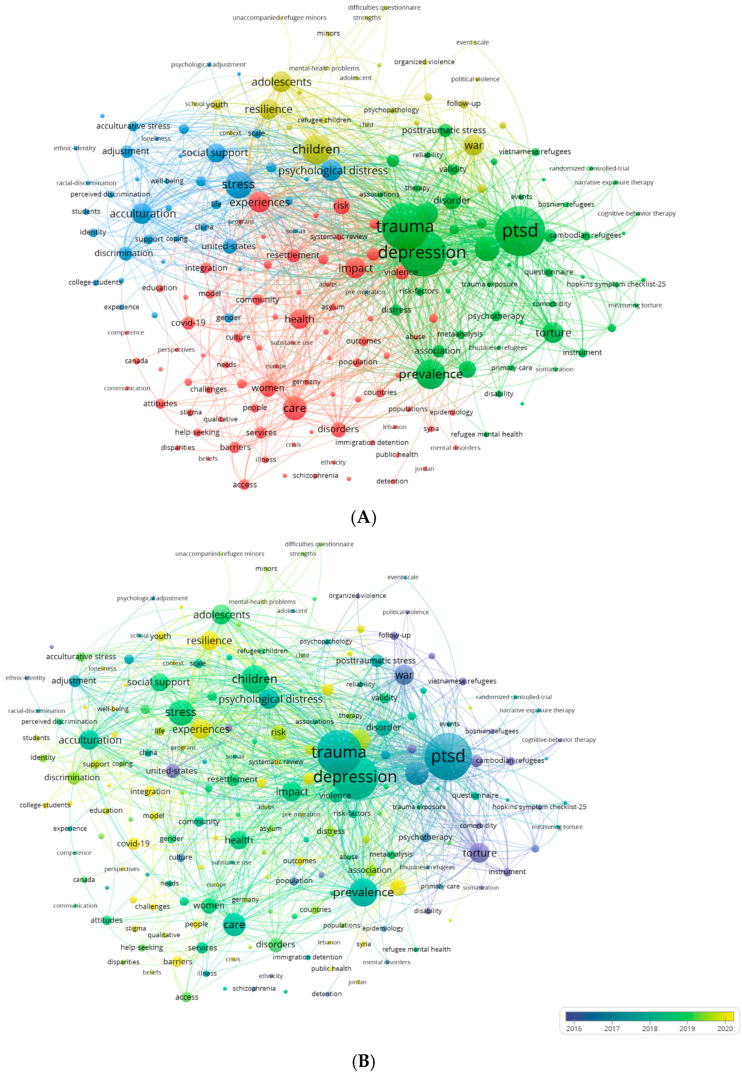
(**A**) Co-occurrence analysis of top keywords. (**B**) Network map of the trend topics according to keywords.

As shown in Figure 6A, we used VOSviewer to build a visualization network map of the 200 keywords in four clusters with co-occurrence: Cluster 1 (red), Cluster 2 (green), Cluster 3 (blue), and Cluster 4 (yellow). The node label is the keyword, and the node size represents its frequency. Links connecting two nodes represent a co-occurring relationship between the keywords. The frequency of occurrence was analyzed based on the average publication year. Figure 6B shows a network map of trending topics according to the keywords used until 2024. Circles near dark blue indicate keywords that were popular earlier (around 2016) and circles closer to bright yellow indicate recently (post-2020) popular keywords.

## 4. Discussion

This study systematically gathered and analyzed bibliometric data pertaining to research on the mental health of international migrants. The analysis delineated prevailing research trends and identified contributing countries, institutions, authors, and relevant keywords associated with the mental health of this population. Previous studies have largely examined clinical or sociological aspects of migrant mental health without quantitatively mapping the field’s structural development or evolution. By providing a visualized overview of intellectual structure and thematic trajectories, this study fills a significant gap in the literature.

Since the first independent research paper was published in 1983, research on international migrants’ mental health has continued to grow over the past 40 years. Particularly in the last five years, research results in this field have shown explosive growth, and the number of papers published every year has exceeded 500, indicating a rapid development trend in this field. In addition, multiple disciplines such as psychiatry, public environmental professions, psychology, and social work are actively participating in research in this field, promoting a multidisciplinary perspective and a more comprehensive understanding of the mental health of international migrants.

According to the countries’ network map, it reveals the global collaborative nature of the research on the mental health of international migrants: the United States and Australia, as prominent migrant-receiving nations, lead international research efforts in this field. Europe has the largest number of migrants [2]. In recent years, European countries have accepted large numbers of refugees based on humanitarian considerations; with Britain and Germany as centers, they formed a close cooperation network on mental health of migrants [28]. Asian countries such as Japan and South Korea reflect the growing emphasis on international migration, especially in the areas of labor migration and cross-border marriages [29,30,31]. This is closely related to the region’s aging population and low fertility rate. Meanwhile, developing countries in Africa and Asia have contributed through collaborative research partnerships, offering critical insights and data on migrants’ countries of origin. These contributions are essential for building a globally comprehensive understanding of migrant mental health, as psychological challenges often stem from their pre-migration environment and experiences [2].

The organizations that were most active in studying the mental health of international migrants were aligned with the core countries of the studies. These highly productive institutions are located in countries with active research, have formed relatively independent and closely linked research networks in their regions and are leading trends in international migrant mental health research. By analyzing the distribution and co-authorship of authors, it became evident that some authors with large nodes and middle locations showed high output and influence in the field. The multiple color-differentiated clusters of collaborations represent different research teams and topics. These multicenter collaborations indicate that the study of the mental health of international migrants has evolved into multiple research directions, each centered on a different core author. Visually, the connections between different clusters were relatively loose. This structure reflects the limited cooperation between different groups in the study of international migrants’ mental health, which may also mean that there is still room for further integration of the field at a multi-team, multi-regional depth.

Mapping a network of common keywords by analyzing their frequency of common keywords in several publications helps determine the internal structure and trends of mental health research on international migrants. By analyzing the co-occurrence of the top 200 keywords, four clusters were established and analyzed as follows.

Cluster 1 (red) mainly focuses on the risk factors experienced by migrants during migration and after settlement and their impact on mental health, particularly the role of violence, gender factors, and epidemics. Violence and traumatic events are more common among migrants, particularly refugees, and these experiences have a significant negative impact on their mental health [32]. Pandemics such as COVID-19 have exacerbated mental health issues among migrant groups, with many migrants facing additional mental health challenges due to lockdowns, social isolation, and economic pressures [33,34].

Cluster 2 (green) focuses on the psychological disorders of migrants and refugees due to traumatic events, such as political conflict, war, violence, and abuse, and their interventions. Migrants, particularly refugees, often experience severe psychological trauma, leading to high rates of PTSD, depression, anxiety, and other mental disorders [35]. The research in this cluster focused on the development and validation of reliable measurement tools to assess the mental health status of migrants and refugees and how effective interventions can improve their mental health of migrants and refugees. It reflects an ongoing effort to build standardized, trauma-informed approaches to mental health care in displaced contexts.

The core theme of Cluster 3 (blue) was acculturation and psychological stress, focusing on the challenges that migrants face when adapting to new cultural and social environments. Migrants may face language barriers, cultural conflict, racial discrimination and social exclusion in their new environment, which together constitute acculturative stress [36,37]. Research in this cluster attempts to reveal how migrants cope with acculturative stress, such as seeking social support, problem-solving, and emotional regulation to facilitate successful integration.

Cluster 4 (yellow) focuses on the mental health and adjustment of migrant and refugee children and adolescents. Children and adolescents are more vulnerable to migration than adults and may be more profoundly affected by traumatic events such as war and family separation [38]. The research in this cluster focuses on the mental resilience of children and adolescents and explores how multilevel supportive environments (such as family, school, and community) can help improve the mental resilience of these groups and promote mental health and social integration.

Research on the mental health of international migrants has undergone a notable shift over the past four decades—from an early focus on traumatic events and psychological disorders to a multidimensional, multi-context comprehensive study. While initial studies focused on traumatic events and their psychological effects, in recent years particularly with the impact of changes in the global social environment and emergencies (such as war and COVID-19), studies have paid more attention to the acculturation, social exclusion, and social integration of migrants in the new environment and have gradually focused on the difficulties of migrants in accessing mental health services and the important role of social support systems in mental health. This shift highlights the field’s increasing responsiveness to real-world challenges and the need for culturally and contextually informed approaches. Importantly, these evolving priorities strongly align with the World Health Organization’s strategic recommendations for promoting migrant mental health and are also consistent with the global development agenda, particularly the United Nations 2030 SDGs. Key strategies such as the integration of services into primary care, the development of community-based support systems, the reduction in structural and linguistic barriers, and the promotion of equitable access to culturally appropriate care for migrant populations contribute to narrowing health disparities, promoting well-being and fostering social inclusion on a global scale [8].

Despite its contributions, this study has several limitations. First, it relied on a single database—Web of Science (WOS) for data collection. Although the WOS is a comprehensive and large-scale, multidiscipline platform, relevant studies from other databases may have been omitted [39]. Moreover, more than 95% of the literature analyzed in this study was published in English, which reflects the dominance of the WOS literature in English but may also lead to language bias, ignoring research published in other languages. These factors may contribute to the underrepresentation of research from countries where English is not the official language or from developing regions. For example, Brazil has received a large number of migrants from neighboring South American countries, especially Venezuela [40], yet this is not significantly reflected in the dataset. This underrepresentation may stem from regional studies being published in local languages (such as Portuguese or Spanish) or in journals that are not indexed in core citation databases such as the WoS. Future research should consider integrating multiple databases and expanding coverage to include regional and multilingual sources.

Second, although the keyword search strategy was designed to ensure conceptual clarity and operational feasibility, it may have inadvertently excluded studies that focused on specific mental health conditions without explicitly using the umbrella term “mental health.” This limitation reflects a necessary trade-off between breadth and specificity in bibliometric search strategies: while the use of a general term like “mental health” allows for the inclusion of multidisciplinary and comprehensive studies, it may inadvertently omit narrowly focused, condition-specific research. Future research could address this by complementing broad umbrella terms with targeted condition-specific keywords to improve coverage while preserving thematic coherence.

Third, bibliometrics analysis mainly adopts quantitative methods and does not evaluate the quality of the literature [26]. As such, variations in study quality may affect the interpretability and reliability of the findings. Incorporating systematic quality assessment frameworks would also enhance the robustness and scientific value of bibliometric analyses. Nonetheless, the present study provides a valuable evidence base for identifying knowledge gaps and guiding future interdisciplinary and policy-relevant research in migrant mental health.

Finally, previous studies have highlighted the significance of gender-specific mental health issues among immigrants, the psychosocial adaptation of elderly migrants, and regional disparities in research [41,42], these themes were relatively underrepresented in the present study, with low frequency and density in the retrieved literature. Given these research gaps, future studies and policy development related to immigrant mental health should urgently pursue more systematic and in-depth exploration of these specific populations and thematic areas.

## 5. Conclusions

International migration is a far-reaching global phenomenon and an important driver of sustainable development [5]. This study systematically analyzed global research on the mental health of international migrants using bibliometric methods. The results revealed a rapid growth in this research field and the gradual formation of international collaboration and multidisciplinary networks. Key research hotspots include PTSD, trauma, depression, and stress, with a notable shift in focus from trauma-centered studies to themes such as social integration and service accessibility. The findings underscore the importance of expanding access to mental health services, developing culturally responsive interventions, and designing support strategies tailored to the needs of vulnerable subgroups. Future research should promote multidisciplinary collaboration to support vulnerable migrant populations, including those in developing countries and marginalized groups, through innovative, evidence-based strategies. Enhancing the breadth and depth of research in migrant mental health is essential for advancing health equity and supporting inclusive, globally informed policy and practice.

## Figures and Tables

**Table 1:** Top 10 countries and regions that conducted research on mental health of international migrants.

Rank	Countries and Regions	Number of Publications	Citation	Total Link Strength
1	United States	2096	54,356	1393
2	Australia	851	24,521	730
3	United Kingdom	778	21,040	1227
4	Germany	625	10,551	707
5	Canada	580	13,856	408
6	Netherlands	338	10,228	759
7	P.R. China	297	6353	287
8	Sweden	259	5179	336
9	Switzerland	245	7520	562
10	Denmark	215	4826	372

**Table 2:** Top five organizations that conducted research on mental health of international migrants.

Rank	Organizations	Country	Publication	Citation	Total Link Strength
1	University of New South Wales	Australia	161	3471	492
2	Toronto University	Canada	142	3899	341
3	Melbourne University	Australia	137	4260	291
4	Karolinska Institutet	Sweden	133	2609	273
5	McGill University	Canada	127	4331	188

**Table 3:** (**A**) Top 10 academic journals that published research on mental health of international migrants. (**B**) Top 10 journals that cited research on mental health of international migrants.

Rank	Academic Journals	Country	Categories	Publication	Citation
(**A**)					
1	International Journal of Environmental Research and Public Health	Switzerland	Public, environmental, and occupational health	189	3070
2	Journal of Immigrant and Minority Health	United States	Public, environmental Occupational health	172	4433
3	BMC Public Health	United Kingdom	Medicine	131	2753
4	Transcultural Psychiatry	United Kingdom	Psychiatry Anthropology	114	2404
5	PloS One	United States	Multidisciplinary	98	1678
6	European Journal of Psych traumatology	United Kingdom	Psychiatry Psychology, clinical	92	2053
7	International Journal of Social Psychiatry	United Kingdom	Psychiatry	85	1615
8	BMC Psychiatry	United Kingdom	Psychiatry	83	2417
9	Frontiers in Psychiatry	Switzerland	Psychiatry	81	1480
10	The Journal of Nervous and Mental Disease	United States	Psychiatry Clinical neurology	80	5271
(**B**)					
1	JAMA—Journal of the American Medical Association	United States	Medicine, general, and internal	21	6487
2	The Journal of Nervous and Mental Disease	United States	Psychiatry Clinical neurology	80	5271
3	Social Science & Medicine	United Kingdom	Public, environmental and occupational health Social sciences Biomedical	70	4742
4	Journal of Immigrant and Minority Health	United States	Public, environmental and occupational health	172	4433
5	International Journal of Environmental Research and Public Health	Switzerland	Public, environmental and occupational health	189	3070
6	Social Psychiatry and Psychiatric Epidemiology	Germany	Psychiatry	68	3048
7	BMC Public Health	United Kingdom	Medicine	131	2753
8	BMC Psychiatry	United Kingdom	Psychiatry	83	2417
9	Transcultural Psychiatry	United Kingdom	Psychiatry Anthropology	114	2404
10	American Journal of Orthopsychiatry	United States	Social work	52	2155

**Table 4:** Top 10 most productive authors in research on mental health of international migrants.

Rank	Authors	Countries/Regions	Publication	Citation
1	Nickerson, Angela	Australia	73	2824
2	Bryant, Richard A.	Australia	66	3618
3	Silove, Derrick	Australia	56	3762
4	Sijbrandij, Marit	Netherlands	44	606
5	Ventevogel, Peter	Switzerland	43	1361
6	Derluyn, Ilse	Belgium	35	934
7	Roberts, Bayard	United Kingdom	35	1202
8	Carlsson, Jessica	Danmark	34	504
9	Cuijpers, Pim	Netherlands	33	465
10	Morina, Naser	Switzerland	33	977

**Table 5:** Top 10 research keywords on mental health of international migrants.

Rank	Keywords	Occurrences	Total Link Strength
1	PTSD	1466	8057
2	trauma	1352	7282
3	depression	1324	7429
4	prevalence	692	3752
5	children	689	3792
6	stress	613	3064
7	symptoms	537	3224
8	Care	516	2200
9	anxiety	458	2713
10	acculturation	433	2332

## Data Availability

The data that support the findings of this study were retrieved from the Web of Science Core Collection. Access to this database requires a subscription and is not publicly available. However, the search strategy, keywords, and analysis procedures are detailed within Section 2 of the manuscript to allow replication.

## Abbreviations

The following abbreviations are used in this manuscript:

PTSD	Post-traumatic stress disorder
WHO	The World Health Organization
IOM	International Organization for Migration
CSV	Comma-Separated Values
COVID-19	Coronavirus Disease 2019
SDGs	Sustainable Development Goals
